# 2094. A prospective multicenter study of HHV-6B genomic DNA and gene transcription in paired bronchoalveolar lavage fluid and blood from HCT recipients

**DOI:** 10.1093/ofid/ofac492.1716

**Published:** 2022-12-15

**Authors:** Joshua A Hill, Yeon Joo Lee, Lisa K Vande Vusse, Hu Xie, E Lisa Chung, Jacob Keane-Candib, Alpana Waghmare, Guang-Shing Cheng, Haiying Zhu, Meei-Li Huang, Geoffrey Hill, Keith R Jerome, Sina A Gharib, Wendy M Leisenring, Danielle M Zerr, Sanjeet S Dadwal, Michael J Boeckh

**Affiliations:** Fred Hutchinson Cancer Center; University of Washington, Seattle, Washington; Memorial Sloan Kettering Cancer Center; Weill Cornell Medical College, New York, New York; University of Washington, Seattle, Washington; Fred Hutchinson Cancer Center, Seattle, Washington; Fred Hutchinson Cancer Center, Seattle, Washington; Fred Hutchinson Cancer Center, Seattle, Washington; Fred Hutchinson Cancer Center; Seattle Children's Hospital, Seattle, Washington; University of Washington; Fred Hutchinson Cancer Center, Seattle, Washington; University of Washington, Seattle, Washington; University of Washington, Seattle, Washington; Fred Hutchinson Cancer Center, Seattle, Washington; Fred Hutchinson Cancer Center, Seattle, Washington; University of Washington, Seattle, Washington; Fred Hutchinson Cancer Center, Seattle, Washington; University of Washington; Seattle Children's Research Institute, Seattle, Washington; City of Hope National Medical Center, Duarte, California; Fred Hutchinson Cancer Center, Seattle, Washington

## Abstract

**Background:**

We previously demonstrated frequent detection of HHV-6B DNA in bronchoalveolar lavage fluid (BALF) and its positive association with mortality in HCT recipients from 1992-2015 with lower respiratory tract disease (LRTD). Whether these findings remain pertinent in contemporary patients, the additive value of testing for viral gene transcription, and the correlation of HHV-6 detection in blood and BALF, are unknown.

**Methods:**

We conducted a prospective study of allogeneic HCT recipients undergoing BAL for LRTD within 120 days of HCT at three cancer centers from 2015-2019. We collected and tested paired blood and BALF for HHV-6B DNA by qPCR and HHV-6B mRNA (U38 and U90 gene transcripts) among DNA positive samples using RT-qPCR. We described the detection of HHV-6B DNA and mRNA in blood and BALF, generated receiver operating characteristic (ROC) curves to determine the ability of BALF HHV-6B DNA detection to predict HHV-6B mRNA detection, and analyzed the association of HHV-6B DNA detection with mortality.

**Results:**

We enrolled 116 allogeneic HCT recipients who underwent 125 BALs. HHV-6B DNA was detected in 45 of 122 BALF (37%) compared to 19 of 124 (15%) plasma samples. Among the 45 BALF samples with HHV-6B DNA detected, either HHV-6B mRNA transcript was detected in 22 (49%) (**Figure 1)**. BALF HHV-6B DNA ≥ 218 copies/ml had an area under the curve of 0.93 for predicting detection of BALF viral mRNA (**Figure 2**). In turn, patients with BALF HHV-6B DNA ≥ 218 copies/mL had increased risk for mortality and death due to LRTD within 60 days after the BAL (**Figure 3**). This association remained after adjustment for age, oxygen use, and steroid use at the time of BAL in a multivariable Cox model (**Figure 3**).

**Figure:**
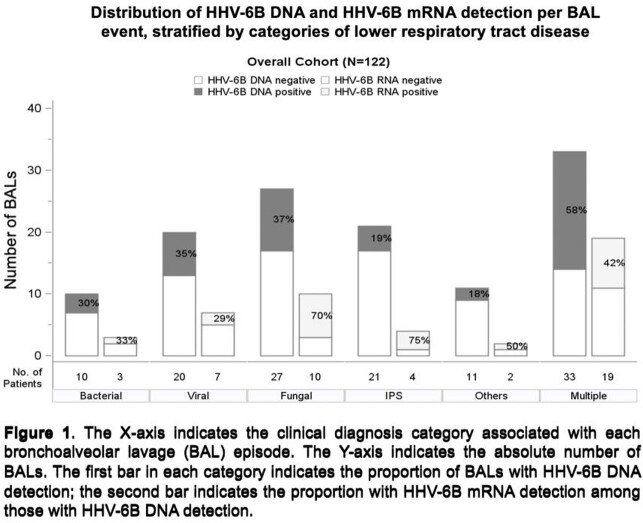

**Figure:**
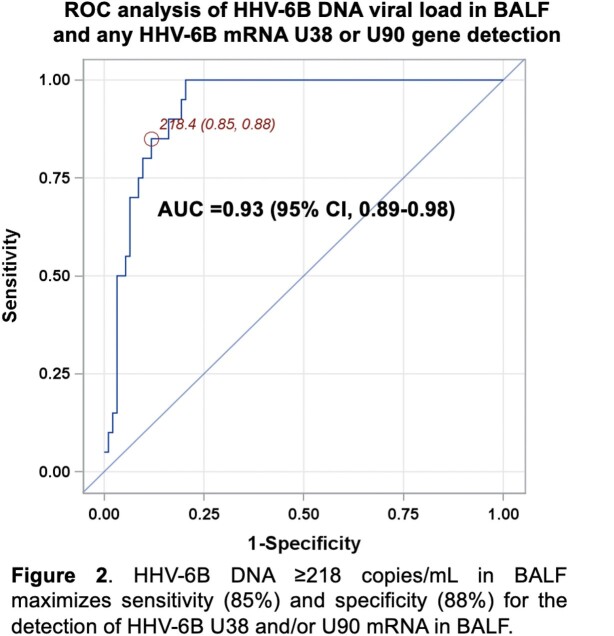

**Figure:**
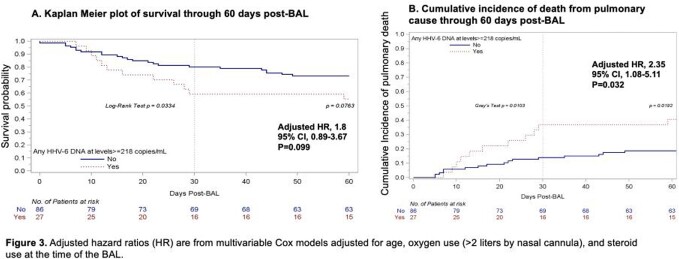

**Conclusion:**

HHV-6B was detected more frequently in BALF than plasma, suggesting compartment-specific reactivation. BALF HHV-6B DNA ≥ 218 copies/mL had high sensitivity and specificity for detection of viral gene transcription in BALF and was associated with increased mortality; this viral load is strikingly similar to the BALF viral load threshold of 251 copies/mL associated with mortality in our prior retrospective study. Together, these data suggest transcriptionally active HHV-6B is a clinically impactful pulmonary pathogen in contemporary HCT recipients.

**Disclosures:**

**Joshua A. Hill, MD**, Allovir: Advisor/Consultant|Allovir: Grant/Research Support|Covance/CSL: Advisor/Consultant|CRISPR: Advisor/Consultant|Deverra: Grant/Research Support|Gilead: Grant/Research Support|Karius: Advisor/Consultant|Karius: Grant/Research Support|Merck: Grant/Research Support|Octapharma: Advisor/Consultant|OptumHealth: Advisor/Consultant|Oxford Immunotec: Grant/Research Support|Pfizer: Advisor/Consultant|Symbio: Advisor/Consultant|Takeda: Advisor/Consultant **Alpana waghmare, MD**, Allovir: Grant/Research Support|Ansun BioPharma: Grant/Research Support|Kyorin Pharmaceutical: Advisor/Consultant|Pfizer: Grant/Research Support|Vir/GSK: Grant/Research Support **Geoffrey Hill, M.D., FRACP, FRCPA**, Applied Molecular Transport: Grant/Research Support|Compass Therapeutics: Grant/Research Support|Generon Corporation: Advisor/Consultant|Heat Biologics: Grant/Research Support|iTeos Therapeutics: Advisor/Consultant|iTeos Therapeutics: Grant/Research Support|Laevoroc Oncology: Grant/Research Support|NapaJen Pharma: Advisor/Consultant|Neoleukin Therapeutics: Advisor/Consultant|Serplus Technology: Grant/Research Support|Syndax Pharmaceuticals: Grant/Research Support **Danielle M. Zerr, MD MPH**, AlloVir: Advisor/Consultant **Sanjeet S. Dadwal, MD, FACP, FIDSA**, AlloVir: Advisor/Consultant|AlloVir: Grant/Research Support|Ansun Biopharma: Grant/Research Support|Aseptiscope: Advisor/Consultant|Aseptiscope: Stocks/Bonds|Astellas: Speaker's Bureau|Cidara: Advisor/Consultant|Gilead: Grant/Research Support|Karius: Grant/Research Support|Merck: Advisor/Consultant|Merck: Grant/Research Support|Merck: Speaker's Bureau|Takeda: Speaker's Bureau **Michael J. Boeckh, MD PhD**, Allovir: Advisor/Consultant|Amazon: Grant/Research Support|Ansun Biopharma: Grant/Research Support|EvrysBio: Advisor/Consultant|Gates Ventures: Grant/Research Support|Gilead Sciences: Advisor/Consultant|Gilead Sciences: Grant/Research Support|GlaxoSmithKline: Advisor/Consultant|GlaxoSmithKline: Grant/Research Support|Helocyte: Advisor/Consultant|Janssen: Advisor/Consultant|Janssen: Grant/Research Support|Kyorin Pharmaceuticals: Advisor/Consultant|Merck: Advisor/Consultant|Merck: Grant/Research Support|Moderna: Advisor/Consultant|Moderna: Grant/Research Support|Regeneron: Grant/Research Support|ReViral: Advisor/Consultant|Symbio: Advisor/Consultant|Takeda: Grant/Research Support|Vir Biotechnology: Advisor/Consultant|Vir Biotechnology: Grant/Research Support.

